# Assessing the impact of academic-practice partnerships on nursing staff

**DOI:** 10.1186/s12912-015-0085-7

**Published:** 2015-05-09

**Authors:** Marjorie L. Pearson, Tamar Wyte-Lake, Candice Bowman, Jack Needleman, Aram Dobalian

**Affiliations:** RAND Corporation, Santa Monica, CA USA; Veterans Emergency Management Evaluation Center (VEMEC), 16111 Plummer St. MS-152, Sepulveda, CA 91343 USA; HSR&D Center for the Study of Healthcare Innovation, Implementation and Policy, North Hills, Los Angeles, CA USA; Department of Health Policy and Management, University of California Los Angeles School of Public Health, Los Angeles, CA USA; University of California Los Angeles School of Nursing, Los Angeles, CA USA

**Keywords:** Nursing education, Partnerships, Nursing Staff, VA Nursing Academy

## Abstract

**Background:**

The ‘spillover effect’ of academic-practice partnerships on hospital nursing staff has received limited attention. In 2007, the Department of Veterans Affairs (VA) created the VA Nursing Academy (VANA) to fund fifteen partnerships between schools of nursing and local VA healthcare facilities. In this paper, we examine the experiences of the VA staff nurses who worked on the units used for VANA clinical training.

**Methods:**

We used survey methods to collect information from staff nurses at all active VANA sites on their characteristics, exposure to the program’s clinical training activities, satisfaction with program components, and perspectives of the impact on their work and their own plans for education (N = 314). Our analyses utilized descriptive statistics and bivariate and multivariate regression.

**Results:**

Results show that staff nurses working on VANA units had moderately high levels of exposure to the program’s clinical education activities, and most reported positive experiences with those activities. The vast majority (80 %) did not perceive the presence of students as making their work more difficult. Among those who were enrolled or considering enrolling in a higher education program, over a quarter (28 %) said that their VA’s participation in VANA had an influence on this decision.

The majority of staff nurses were generally satisfied with their experience with the students. Their satisfaction with the program was related to the level or dose of their exposure to it. Those who were more involved were more satisfied. Greater interaction with the students, more information on the program, and a preceptor role were all independently associated with greater program satisfaction.

**Conclusions:**

Our study suggests that academic-practice partnerships may have positive spillover effects on staff nurses who work on clinical education units. Further, partnerships may be able to foster positive experiences for their unit nurses by focusing on informing and engaging them in clinical training activities. In particular, our results suggest that academic-practice partnerships should keep unit nurses well informed about program content and learning objectives, encourage frequent interaction with students, involve them in partnership-related unit-based activities, and urge them to become preceptors for the students.

**Electronic supplementary material:**

The online version of this article (doi:10.1186/s12912-015-0085-7) contains supplementary material, which is available to authorized users.

## Background

Training in a clinical facility, whether under the guidance of a clinical faculty member, a formal preceptor, or a member of the nursing staff, is a vital part of the baccalaureate nursing curriculum. Generally, a clinical faculty member brings students to a clinical site, but once there, the amount of interaction with nurses on staff becomes highly variable. Nursing preceptors are generally defined as experienced nursing staff members, who hold a dual role of carrying out their usual patient care responsibilities while orienting new employees and supervising and guiding students, all on a one-to-one basis [1]. However, during the clinical experience, other nurses on staff also interact with students, both formally and informally, and are likely to be impacted by their presence [2, 3]. Most nursing staff are involved in some way in guiding students on their units, from taking care of the same patient to observing them at a distance, in hospitals that host students.

Academic-practice partnerships have become increasingly more prevalent in nursing education [4, 5]. Some such partnerships, such as the Veterans Affairs Nursing Academy (VANA) created by the Department of Veterans Affairs (VA), have focused especially on involving VA staff nurses in clinical education activities. Beginning in 2007, the VA funded 15 pilot partnerships between schools of nursing (SONs) and local VA healthcare facilities throughout the country to help address nursing shortages [6]. The fifteen partnerships were funded in three waves, four in 2007, six in 2008, and five in 2009. The VA provided the partnerships with grants to support salaries for additional faculty positions, based either at the VA Medical Center (VAMC) or partnering university, to provide clinical, classroom, simulation lab and/or other kinds of instruction. One of the program’s primary goals was to increase recruitment and retention of VA facility nurses by enhancing their roles in nursing education. As such, the majority of VANA faculty who took on the role of clinical nurse faculty were VA nurses [5] who were already well-integrated into the VA hospital environment. Evaluation findings suggest that these nurses were quite satisfied with their experience in the VANA program [7]. In this paper, we examine the experience of the VANA unit nurses who did not take on a faculty role.

Little is written about the impact of academic-practice partnerships, such as VANA, on the staff nurses in these clinical training environments. There has been some focus on the ‘spillover effect’ of these partnerships on nursing staff, but that has been mostly limited to exposure to new career options, such as teaching and research, as well as stimulating a desire to further one’s education [4, 5, 8]. The effects on staff nurses working in a clinical training environment more generally, such as role ambiguity, work overload, and lack of recognition, have been described, but primarily from the formal preceptor’s perspective. Key issues such as perceptions of benefits (e.g. increased professional growth), rewards (e.g. professional recognition), and needed support systems (e.g., leadership involvement) also have been explored for preceptors [1, 9–17]. Apart from nursing faculty and students, who are the obvious targets of academic-practice partnerships, nursing staff, in both the formal and informal roles of preceptor, engage with both faculty and students in the clinical environment to a great, but largely unmeasured, extent.

The purpose of our study was to characterize participating nursing staff, their VANA-related roles and responsibilities, and their satisfaction with certain aspects of participation. We also examined the relationship between exposure to students and satisfaction with the program.

Our research questions (RQs) were:

1. To what extent were the staff nurses on VANA units exposed to the clinical education activities on their units?
2. Did these clinical education activities impact their own work and education plans, in their view?
3. How satisfied were they with the clinical education components of their work environment?
4. Did the extent of their exposure to the VANA program relate to their perception of the program’s impact and to their sense of satisfaction with these clinical education components?

## Methods

As part of the larger national VANA evaluation project, we surveyed unit nursing staff who were working on units where VANA students were placed. The survey, fielded in 2012 at all VANA sites, was designed to gather information on a number of dimensions of the staff nurses’ experience working on units which hosted these clinical training programs. The 30-item survey was designed by the project team and consisted of close-ended questions with some opportunity for open comments (See Additional file 1). Given the paucity of literature on the experience of staff nurses in academic-practice partnerships, we were not able to find an existing, validated instrument appropriate for our evaluation. Additionally, a number of our evaluation questions were not routine questions, but were specific to the VANA program. We therefore were in a position where we had to develop the instrument ourselves. In part to inform the survey development, we conducted focus groups of the non-faculty unit nurses. As part of the larger National VANA Evaluation, we fielded two additional surveys, one of faculty and one of students. Although those results are not reported here, the results and experiences of those surveys also helped to inform the development of the staff nurse survey. Because the questionnaire was tailored to a specific intervention, it could not be tested in a separate sample; however, we pre-tested it in a subset of our sample (four sites) in 2010, and subsequently refined the survey after this initial pilot. All registered nurses identified to be on a VANA unit at the time when VANA students were present were asked to complete the survey. The National Evaluation Team sent packets of paper surveys with pre-addressed return envelopes to the VA Program Director, who distributed the packets to the VANA unit managers. After explaining and distributing the survey to the identified staff nurses at a unit staff meeting, the unit managers left the room, and the staff nurses completed the surveys and put them into the pre-addressed return envelope. The last nurse to complete the survey sealed the envelope and took it to the unit manager who mailed it back to the National Evaluation Team. The survey’s cover page explained the purpose of the survey, its confidentiality and privacy provisions, and the fact that participation was “strictly voluntary”. Completion and return of the survey was accepted as consent. The VA Greater Los Angeles Healthcare System Institutional Review Board approved this study.

The survey included questions on the staff nurse characteristics and their exposure to the clinical training program activities on their unit, as well as the outcomes of interest. Staff nurse characteristics included age, gender, years in nursing, years at a VA facility, highest degree, current position, and full-time or part-time status.

Measures of their exposure to clinical program training activities were derived from questions on the frequency of student presence on their unit, the frequency of their interaction with the students when present, the extent to which they felt informed about the program, and their status as a preceptor of the students. The survey also included a question on the staff nurses’ perception of change in activities designed to foster the use of evidence-based practice (EBP) on their unit since the start of VANA. “Evidence-based practice” was defined as “integrating the best available research evidence with…patient preferences, clinician skill level, and available resources to make decisions about patient care” [18]. They were asked to consider such activities as in-services, team meetings, distribution of articles, and journal clubs. Perceived increase (moderate or substantial) is considered exposure to successful program implementation. All exposure variables were constructed to reflect the extent of nurse exposure (i.e., the dose) to allow for “dose–response” analyses. Responses to the question on the frequency of interaction with nursing students, for example, were categorized into high, medium, or low frequency. The exposure measures were self-reported, and survey respondents were allowed to use their own definition of preceptor status.

The outcome measures were derived from questions asking them to assess the students’ impact on the difficulty of their work, the program’s influence on their own education plans, and their satisfaction with program components, such as the professional recognition they received from working with students, their personal reward from working with students, and the clinical expertise of the clinical faculty. The terms *clinical faculty* and *clinical instructor* are used interchangeably in this manuscript.

In this paper, we present analyses from the survey data collected in 2012. Descriptive statistics were used to summarize the characteristics of participating nursing staff, their VANA-related roles and responsibilities, and their satisfaction with certain aspects of participation. We used bivariate and multivariate regression to examine the relationship between the exposure variables (and the dose of exposure) and the outcome variables. All statistical analyses were performed using Stata 12 [19].

## Results

The VA Program Directors identified a total of 66 VANA units (mean = 5 units/VA; range = 2–10) from the thirteen existing partnerships at the time of the study. Table 1 highlights key characteristics of the participating sites. Most Program Directors specified day shifts as the shift exposed to VANA students; although three directors reported placing students on other shifts as well. Altogether, Program Directors identified 758 staff nurses assigned to units on shifts when VANA students were present. Surveys were returned by 340 staff nurses from 57 units for an overall response rate of 45 % (range: 15-91 % for individual VAs). Response rate by partnership cohort was variable. Twenty six returns from LVNs/LPNs were excluded, leaving a total of 314 returns from RNs. The RN data are reported here.

**Table 1:** Characteristics of partnering institutions as reported at the time of the study

Educational Institution	Type	General enrollment	# students pre VANA	VA	# Nursing school affiliations	# RNs prepared at BSN level	Proportion of total response rate (N = 314)
**COHORT 1**							28 %
University of Utah	Public	28,025	128	VA Salt Lake City HCS	5	205	
Fairfield University	Private	~5000	321	VA Connecticut HCS	4	†	
University of Florida	Public	>49,000	700	North Florida/South Georgia Veterans Health System	2	†	
San Diego State University	Public	35,832	†	VA San Diego HCS	4	†	
**COHORT 2**							60 %
University of South Florida	Public	1828	315	James A. Haley Veterans Hospital	5	450	
Medical University of South Carolina	Public	2532	50	Ralph H. Johnson VAMC	2	122	
Loyola University Chicago	Private	15,000	427	Edward Hines Jr. VA Hospital	3	340	
University of Detroit Mercy	Private	6000	933	Aleda E. Lutz VAMC	10	98	
Saginaw Valley State University	Public	9500	320	John D. Dingell VAMC	4	56	
University of Oklahoma	Public	>30,000	96	Oklahoma City VAMC	2	154	
Rhode Island College	Public	9000	141	Providence VAMC	8	91	
**COHORT 3**							12 %
University of Hawaii at Mãnoa	Public	20,169	112	VA Pacific Islands HCS	2	65	
University of Alabama at Birmingham	Public	16,874	427	Birmingham VAMC	16	104	

HCS: Healthcare System

VAMC: VA Medical Center

† Unspecified

### Respondent characteristics

The staff nurses were well distributed across age ranges and years of nursing experience, as shown in Table 2. Nearly three-quarters were over 35 years old (72.1 %) and over half had 10 or more years in nursing (54.1 %). A quarter had worked for 10 or more years in the VA (24.9 %). A sizable minority, however, were relatively new to their positions; more than a quarter had less than 5 years in nursing. Most were women (81.9 %) with Bachelor-level degrees or higher. Almost all were staff nurses (93.9 %) and worked full-time (94.2 %).

**Table 2:** Respondents’ characteristics

Characteristic	Number	Percent
Age (total N = 290)^a^		
Under 25	27	9.3
26–35	54	18.6
36–45	75	25.9
46–55	82	28.3
Over 55	52	17.9
Gender (total N = 298)		
Female	244	81.9
Male	54	18.1
Years in nursing (total N = 314)		
Less than 1 year	8	2.6
1 to less than 5 years	80	25.5
5 to less than 10 years	56	17.8
10 to less than 20 years	78	24.8
20 or more	92	29.3
Years at a VA facility (N = 314)		
Less than 1 year	25	8.0
1 to less than 5 years	123	39.2
5 to less than 10 years	88	28.0
10 to less than 20 years	43	13.7
20 or more	35	11.2
Highest degree^†^ (N = 301)		
Doctoral degree	0	0
Master’s degree	30	10.0
Bachelor’s degree	195	64.8
Associate’s degree	76	25.3
Current position (N = 314)		
Staff RN	295	93.9
CNS/CNL	3	1.0
Nurse manager	13	4.1
Nurse practitioner	1	0.3
Nurse educator	1	0.3
Other	1	0.3
Full-time or part-time (N = 313)		
Full-time	295	94.2
Part-time	18	5.8

^a^there were 314 returned surveys, but the N in parentheses reflects the number of respondents for the particular question

^†^Includes both nursing and non-nursing degrees

### RQ 1: exposure to the VANA program

Table 3 provides information on the nature of these staff nurses’ exposure to VANA program activities and the roles they played. Since these respondents were selected from VANA units, it is not surprising that all but 2 % reported that nursing students were present on their units during their shifts some of the time. Almost two-thirds (62.5 %) said students were present about half or more of the time on their shifts. An even higher proportion (70.8 %) said they interacted with nursing students about half or more of the time. While most were frequently exposed to students, fewer staff nurses (28.1 %) were well-informed about the VANA program. Many (52.7 %) reported they saw a moderate or substantial increase in EBP activities on the unit since the program started. Over half (55.5 %) of the staff nurses served as preceptors during the VANA period, with 27.6 % performing this role for the first time. Almost half (47.1 %) received preceptor training during the VANA period.

**Table 3:** Respondents’ exposure to VANA program

VANA Exposure	Number	Percent
Nursing students present on unit (N = 296)^a^		
All of the time	4	1.4
Most of the time	75	25.3
About half of the time	106	35.8
A little of the time	105	35.5
None of the time	6	2.0
Interacted with students when present (N = 298)		
All of the time	24	8.1
Most of the time	111	37.3
About half of the time	76	25.5
A little of the time	76	25.5
None of the time	11	3.7
Informed about VANA program (N = 302)		
Well informed	85	28.2
Somewhat informed	140	46.4
Not very informed	77	25.5
EBP activities on unit since VANA start (N = 298)		
Noticed no to slight increase	141	47.3
Noticed moderate to substantial increase	157	52.7
Preceptor role (N = 297)		
Not current preceptor	132	44.4
Current preceptor	165	55.6
Preceptor training since VANA start (N = 242)		
Yes	114	47.1
No	124	51.2
No such program at this facility	4	1.7

^a^there were 314 returned surveys, but the N in parentheses reflects the number of respondents for the particular question

### RQ 2: Perceptions of program impact

As shown in Table 4, the staff nurses varied considerable in how they perceived the effects of the VANA program on their work, educational mobility, and satisfaction. Equal proportions of the staff nurses saw the nursing students as making their work either less or more difficult (20 % each way). The majority (54.8 %) did not think the nursing students impacted the difficulty of their work either way.

**Table 4:** Respondent outcomes

Outcomes	Number	Percent
Impact of nursing students on their work (N = 291)^a^		
Made work less difficult	60	20.6
Did not impact the difficulty of work	172	54.8
Make work more difficult	59	20.3
Influenced toward educational mobility (N = 285)		
Pursing higher education and		
● VANA participation influenced this decision	45	15.8
● VANA participation did not influence this decision	114	40.0
● Not pursuing higher education	126	44.2
Very satisfied or satisfied with experience with nursing students re:		
Aptitude of students (N = 300)	198	66
Clinical expertise of instructors (N = 299)	197	66
Teaching ability of instructors (N = 299)	191	64
Preceptor-to-student ratio (N = 297)	190	64
Personal reward from working with students (N = 298)	188	63
Instructor involvement in teaching students while on the unit (N = 299)	185	62
Support from supervisors/colleagues to work with students (N = 298)	182	61
Information provided to you about student’s learning objectives (N = 297)	175	59
Instructor-to-student ratio (N = 293)	161	55
Time available for you to work with students (N = 297)	160	54
Professional recognition for working with students (N = 297)	143	48

^a^there were 314 returned surveys, but the N in parentheses reflects the number of respondents for the particular question

More than half of the staff nurses (55.8 %) were enrolled or considering enrolling in a higher education program within the next two years. Twenty-eight percent of these 55.8 % of staff nurses – or 15.8 % of all staff nurse respondents -- said that their VA facility’s participation in VANA had an influence on their decision to pursue this higher degree or enroll in this educational program.

### RQ 3: Satisfaction with clinical education components

Table 4 also shows the nurses’ satisfaction with various programmatic aspects of their experience with students over the past year. Since all of the staff nurse respondents worked on VANA units, their responses primarily reflect their experience with VANA students and the VANA program. In general, the majority of staff nurses were satisfied with the different aspects of their experiences with the students. The least number (48 %) were satisfied with the professional recognition they received for working with the students. On the other hand, most (66 %) were satisfied with the aptitude of the students and the clinical expertise of the instructors.

### RQ 4: Relationship of exposure to perceptions of impact

None of the VANA exposure variables in Table 3, nor any of the staff nurse characteristics in Table 2, were related in bivariate regressions (not shown) to the nurses’ perceptions of the program’s impact on their work or education goals.

These analyses also revealed no significant relationships between any of the staff nurses’ characteristics and their satisfaction with student clinical training on the VANA units. They did suggest, however, that the nurses’ satisfaction was correlated with their exposure to the clinical training program, particularly with the extent to which the nurses were informed about details of the program (e.g., learning objectives), frequency of students present on the unit, frequency of the staff nurses’ interactions with students when present, increase in EBP activities observed, and preceptor roles.

Table 5 shows the results of multi-variable regression analyses of the relationship of these exposure variables and the nurses’ satisfaction with the program. (Frequency of students present on the unit was dropped from the model due to its high correlation with frequency of their interaction with students when present on the unit). Table 5 shows that nurses’ exposure to the VANA program in any of these areas (information, interaction, EBP activities, or preceptorship) was significantly associated with a higher degree of satisfaction with the specified aspects of the program, after taking into account their exposure in the other areas. The extent to which staff nurses felt they were informed about the program was significantly associated with their satisfaction with all aspects of the program, except how satisfied they were with the aptitude of the students. The odds of satisfaction with the teaching ability of the instructors, the clinical expertise of the instructors, their personal reward from working with students, and the professional recognition they received from working with students were particularly high (>4.0) for those nurses who felt well informed about the program, as compared with those who were not very well informed. The odds of satisfaction with most program aspects also were significantly higher for staff nurses who felt somewhat informed as compared with not very informed about the program, although not as high as the odds for the staff nurses who felt well informed. A dose response is suggested between the degree that staff nurses were informed about the program and their satisfaction with its various components; the more information they had about the VANA program, the more aware they were of program particulars, such as the quality of the instructors.

**Table 5:** Multi-variate relationship between staff nurses’ program exposure and program satisfaction

	Satisfaction with Program Components (Odds Ratios) (1):										
	Personal Reward	Prof Recognition	Instructor Clinical Expertise	Instructor Teaching Ability	Instructor Involvement	Info Provided	Time Available	Support Received	Preceptor -Student Ratio	Instructor -Student Ratio	Aptitude of Students
Exposure:											
Informed about VANA program (2):											
Well informed	4.21***	4.03***	4.76***	4.94***	2.81**	n/a	3.68***	2.85**	3.07**	2.74**	2.62
Somewhat informed	1.81°	2.12*	3.30***	2.50*	2.02*	n/a	2.89**	2.38*	2.98**	2.38*	1.74
Frequency of interaction with students (3):											
High frequency	4.65***	3.03***	3.52***	3.32***	2.35**	5.27***	4.26***	2.27*	1.99*	1.95*	2.72**
Medium frequency	2.85**	2.53**	3.65**	4.11**	2.41*	2.17°	3.76***	3.35**	2.00	2.67*	4.36***
Noticed an increase in EBP activities on unit (4)											
Moderate to substantial	1.88*	1.84*	3.63***	3.59***	2.31**	2.24**	1.31	2.19**	1.92*	1.67°	2.04*
Precepts students (5)											
Yes	2.43***	1.44	2.51**	2.48**	2.33**	3.06***	2.05*	2.12*	3.62***	2.01*	1.75°

(1) The experiences were specified as:

• Personal reward from working with students

• Professional recognition you received for working with students

• Clinical expertise of instructors

• Teaching ability of instructors

• Instructor involvement in teaching students while on the unit

• Information provided to you about students’ learning objectives

• Amount of time available for you to work with students

• Support you received from supervisors/colleagues to work with students

• Preceptor-to-student ratio

• Instructor-to-student ratio

• Aptitude of students

(2) Extent to which staff nurse felt informed about the VANA program: well informed, somewhat informed, not very informed. Not very informed is the omitted variable

(3) Interacted with nursing students over the past year when present during shift: most or all of the time (defined as high frequency), about half of the time (medium frequency), or a little or none of the time (low frequency). Low frequency of interaction with students is the omitted variable

(4) Noticed a moderate or substantial increase in activities designed to foster the use of evidence-based practice on unit since the start of VANA (dichotomous variable)

(5) Currently performs role of preceptor (dichotomous variable) nn

° p < 0.10, * p < 0.05, ** p < 0.01, *** p < 0.001

Staff nurses’ frequency of interaction with nursing students over the past year also is significantly related to their satisfaction with most aspects of the program. With this exposure variable, the nurses who reported the middle dose (medium frequency of interaction with student) had higher odds of satisfaction with many program aspects than did the top dose (high frequency of interaction with nursing students), when compared to the nurses who reported the bottom dose (low frequency of interaction). The odds of being satisfied with their personal records from working with students and the amount of time available to them to work with students, in particular, were high (>4.0) for nurses who report high frequency of interaction with the nursing students, as were the odds of being satisfied with the teaching ability of the instructors and the aptitude of the students for nurses who report medium frequency of interaction with the students.

Additionally, the staff nurses who were exposed to a moderate or substantial increase in EBP activities on the VANA unit and those who precepted nursing students had significantly higher odds of program satisfaction than did those who were not so exposed, although the odds ratios tended to be a little smaller than those for the other exposure variables. In particular, the nurses who noticed a moderate to substantial increase in EBP activities on the unit had higher odds (>3.0) of being satisfied with the instructors’ clinical expertise and teaching ability. The preceptors had higher odds (>3.0) of being satisfied with the preceptor-to-student ratio.

## Discussion

In this study, we sought to examine the experiences of the nurses who worked on the VANA clinical training units, but who did not have formal faculty roles. Understanding the potential impacts of academic-practice partnerships on the work environment for these nurses, as well as the educational gains for the student nurses, is imperative to optimizing academic-practice partnerships’ goals.

There are several key findings from this study. First, staff nurses working on VANA units reported moderately high levels of exposure to clinical training activities, and most interacted with VANA students a sizable proportion of the time that they were caring for patients on the units. Simultaneously, over half of respondents noticed a moderate or substantial increase in activities designed to foster evidence-based practice on the units. These findings suggest that the clinical training activities were an integral part of the work environment of the non-faculty nursing staff on the VANA units.

Secondly, our results suggest that most nurses did not view their interactions with VANA students as burdensome. Although there is evidence that nursing staff can view the preceptor role as a burden [12, 14, 20], the vast majority of these nurses did not see students as making their work more difficult. A number reported direct benefits. Among those who were enrolled or considering enrolling in a higher education program, over a quarter said that their VA’s participation in VANA had an influence on this decision. This last finding suggests that academic-practice partnerships may create a gateway for nursing staff to advance their education. In turn, this opportunity has the potential to contribute to the realization of the Institute of Medicine’s recommendations that healthcare organizations encourage and ensure that their nurses engage in lifelong learning [21].

Thirdly, the majority of staff nurses on VANA units were satisfied with the features of the clinical training program. Their high satisfaction with the clinical expertise of the instructors may relate to the fact that many of the VANA-supported clinical faculty were recruited directly from the VA facility [7]. The literature suggests that preceptors prefer working with clinical faculty who have had experience either on the same unit or in the same hospital, because the familiarity tends to minimize disruptions to work flow and create a better understanding of staff nurse expectations [17, 22]. The relatively low level of satisfaction with the professional recognition received for working with students may suggest a clear opportunity for improving satisfaction. Given that professional recognition is considered an important benefit of the preceptor role [9, 10, 12, 15], it might be effectively leveraged to benefit all staff nurses who work with students, whether in a formal preceptor role or not.

Fourth, the nurses’ satisfaction with the VANA program was proportional to the level, or ‘dose’, of their exposure to the VANA program and its students. This finding is consistent with the emphasis on nursing staff engagement in another academic-practice partnership [23]. It also suggests that VANA offered some rich opportunities to the VA facilities to enhance the professional experience of their nursing staff. In our study, greater interaction with the students, more information about the program, involvement in EBP activities, and a formal preceptor role were all independently associated with greater program satisfaction. Many have found that various types of support are essential to facilitating nurse satisfaction in the preceptor role. While previous reports have indicated that preceptors desire information about a clinical training program and its students [11, 14, 15, 17], our finding suggests that other staff nurses on the units may have shared their colleagues’ desire to be well informed about the VANA program. The higher satisfaction of the VANA preceptors may be related to the greater role clarity that comes with being a formally designated preceptor. Others have found that nurses reported increased stress and role ambiguity when leadership did not clearly delineate their roles [9, 14, 17, 24]. Those respondents in our study who noted increased involvement of the clinical faculty were also more satisfied with the program, which is consistent with what others have reported [9, 11, 12, 15]. The more involved the faculty are on the unit, the less the students attached to that faculty are seen as a burden. This suggests that increased participation would also provide additional opportunities for expert clinical faculty to act as role models for the students and staff. Similarly, those respondents who noticed a moderate to substantial increase in EBP activities on the unit also were more satisfied with the program.

### Limitations

While every attempt was made to faithfully characterize the impact of the VANA program on the professional experiences of the staff nurses who were affected by it, there were limitations to these findings. These results are limited to academic-practice partnerships implemented within the VA. The unique characteristics of VA facilities may not generalize well to private sector partnerships. Further research is needed to establish if staff nurses in all types of clinical training settings share similar experiences.

We attempted to maximize the representativeness of the staff nurses who worked with VANA students, although those who were not present at the staff meetings when the surveys were distributed (e.g., those taking sick leave that day) did not have the opportunity to participate. While the overall response rate was fairly high for a survey, the rate across units within facilities was somewhat uneven allowing some nurses to be under represented when data were aggregated.

When asked questions about VANA students, respondents may not have always differentiated between VANA students and nursing students from other universities and/or community colleges, despite clear differences in the uniforms they wore. Also, respondents were allowed to self-identify as preceptors, which may have caused us to draw false conclusions about their answers. Because the term is used so broadly in the professional vernacular though, we chose to leave it undefined to capture the perceptions of those nurses who were put into instructional roles, despite whether they were formally or informally designated.

## Conclusions

Our evaluation findings suggest that the experience of working in a VANA clinical training environment was positive for most staff nurses. We found

- moderately high levels of exposure to the student nurses, EBP activities, and preceptor roles among unit nurses in this academic-practice partnership;
- minimal report of being burdened by the students’ presence;
- modest impact on their own motivation to pursue further education;
- moderately high levels of satisfaction VANA program components; and
- a significant relationship between the degree of their involvement in VANA activities and the extent of their satisfaction with the programs. (The staff nurses who were more involved with VANA were more satisfied.)

Further research is needed to clarify the relationship of program implementation to these perceptions and experiences, as well as the applicability of these findings to other academic-practice partnership programs. If these findings and their wider generalizability are supported, they may imply strategies for further engaging and benefiting the staff nurses involved. Future partnerships may be able to foster positive experiences for nurses working on clinical training units by maximizing their information about the program’s content and learning objectives, encouraging frequent interaction with students, involving them in partnership-related unit-based EBP activities, and engaging and training more of them as preceptors.

## Additional file

Additional file 1:
**VA Nursing Academy Staff Nurse Survey 2012.**
