# Supplementary material for: Assessing the impact of academic-practice partnerships on nursing staff
Source: BMC Nurs. 2015 May 9;14:28. doi: 10.1186/s12912-015-0085-7 (PMC4430985; doi:10.1186/s12912-015-0085-7)
Supplement: Additional file 1: — VA Nursing Academy Staff Nurse Survey 2012. [file 12912_2015_85_MOESM1_ESM.pdf]

VA Nursing Academy Staff Nurse Survey 2012

Welcome to the 2012 VA Nursing Academy Staff Nurse Survey!

About this survey

This survey is being conducted as part of the national evaluation of the VA Nursing Academy (VANA), a program that partners VA facilities with nearby university nursing schools. Over the past year, nursing students participating in VANA have done their clinical rotations on selected units at your VA facility. In this survey, we are seeking to understand how VANA has affected participating units. Questions ask about your experiences with nursing students, and the impact that they have had on you and your work. The information we obtain in this survey is critical to our ability to share lessons learned from this partnership with other partnerships that are currently engaged in or plan to participate in VANA in the future.

About your participation

This survey is strictly voluntary; your participation decision will have no adverse effect on you professionally or personally. However, it is essential that we collect information from staff nurses, such as yourself.

Confidentiality and privacy

Your individual survey responses will be kept confidential and results will only be reported in aggregate. No identifying information will be collected with these surveys. Confidentiality will be maintained by keeping all electronic data on password-protected computers and hard copy documents in locked file cabinets. Only evaluation team staff will have access to these data.

How to answer this survey

- We estimate that it will take about 15 minutes to complete this survey, depending on your answers.
- Questions in this survey are based on your judgments and best estimates. You will not need to reference any documents in order to respond.
- Questions that ask about a specific timeframe (e.g., the past year) can be approximated.
- Several questions have skip instructions. Please read and follow these instructions carefully.
- We value your feedback and have provided space for comments at the end of the survey.
- If you have any questions or concerns, please contact the National Evaluation Project Director, Tamar Wyte, DPT, MPH, by phone at (818) 891-7711 x7940 or by email at tamar.wyte@va.gov

We thank you for your effort!

The VA Nursing Academy National Evaluation Team

We would like to start with a few general questions about your work.

1. **How many total years have you worked at any VA facility? Include all VA work, including your current VA position.**
   (Choose one)
   - ☐ Less than 1 year
   - ☐ 1 to less than 5 years
   - ☐ 5 to less than 10 years
   - ☐ 10 to less than 20 years
   - ☐ 20 years or more

2. **How many total years have you worked in nursing? Include all aspects of nursing (e.g., patient care, research, administrative, work as nursing aide/tech, LVN/LPN).**
   (Choose one)
   - ☐ Less than 1 year
   - ☐ 1 to less than 5 years
   - ☐ 5 to less than 10 years
   - ☐ 10 to less than 20 years
   - ☐ 20 years or more

3. **How long have you worked at [VA]?**
   (Choose one)
   - ☐ Less than 1 year
   - ☐ 1 to less than 5 years
   - ☐ 5 to less than 10 years
   - ☐ 10 to less than 20 years
   - ☐ 20 years or more

4. **What is the title of your current position at [VA]?**
   (Choose one)
   - ☐ Staff RN
   - ☐ Staff LVN/LPN
   - ☐ CNS/CNL
   - ☐ Nurse manager
   - ☐ Nurse practitioner
   - ☐ Nurse educator
   - ☐ Other (Specify): ____

5. **Do you currently work part time or full time at [VA]?**
   (Choose one)
   - ☐ Part time (less than 36 hrs/wk)
   - ☐ Full time (36 hrs/wk or more)

6. **Have you worked on [Unit] within the past month?**
   (Choose one)
   - ☐ Yes ➡ Please answer about this unit for questions that ask about "your unit."
   - ☐ No ➡ Specify your current primary unit (below) and answer about that unit for questions that ask about "your unit."

     Current primary unit:____

7. **How long have you worked on your unit?**
   (Choose one)
   - ☐ Less than 1 month
   - ☐ 1 to less than 3 months
   - ☐ 3 to less than 6 months
   - ☐ 7 to less than 12 months
   - ☐ 1 year or more

This next question asks about evidence-based practice* at your facility.

8. **Since [VANAStart], to what extent have you noticed an increase or decrease in activities designed to foster the use of evidence-based practice on your unit and throughout the nursing service as a whole at your facility?**
   Include activities such as in-services, team meetings, distribution of articles, and journal club. Please answer separately about your unit and the nursing service.

	Noticed a substantial *increase* in activities	Noticed a moderate *increase* in activities	Noticed a slight *increase* in activities	Did not notice an increase or decrease	Noticed a slight *decrease* in activities	Noticed a moderate *decrease* in activities	Noticed a substantial *decrease* in activities
	Choose one on each line						
Your unit	☐	☐	☐	☐	☐	☐	☐
The nursing service as a whole at your facility	☐	☐	☐	☐	☐	☐	☐

*By 'evidence-based practice' we mean "integrating the best available research evidence with...patient preferences, clinician skill level, and available resources to make decisions about patient care." (Ciliska, et. al. (2001) *Resources to Enhance Evidence-based Nursing Practice.* Evidence-Based Practice, November 12(4):520-528)

We would now like to know about preceptorships at your facility.

9. **Which of the following best describes your experience as a nursing preceptor at this facility?**
   (Choose one)
   - ☐ You were a preceptor prior to [VANAStart], but are not currently a preceptor
   - ☐ You were a preceptor prior to [VANAStart], and are currently a preceptor
   - ☐ You were not a preceptor prior to [VANAStart], but are currently a preceptor
   - ☐ You have never been a preceptor at this facility/
     no nursing preceptors at this facility ➨ **GO TO Question 11**

10. **Since [VANAStart], did you enroll in a preceptor training program at your facility?**
    (Choose one)
    - ☐ Yes
    - ☐ No
    - ☐ No such program at this facility

These next questions are about your interaction with nursing students on your unit.

11. **During the past year, were you interacting with <u>VANA</u> students assigned to your unit?** If you cannot distinguish VANA students from non-VANA students (i.e., traditional [University] students), check "not sure."
    (Choose one)
    - ☐ Yes
    - ☐ No ➨ **GO TO Question 13**
    - ☐ Not sure ➨ **GO TO Question 13**

12. **How do you identify students who are part of the VANA program?**
    (Choose all that apply)
    - ☐ Students are introduced to you by VANA instructors
    - ☐ Students introduce themselves to you
    - ☐ Students wear a uniform, badge, or pin that identifies them
    - ☐ Student names are distributed or posted
    - ☐ Other means of identification (Specify): ____________________________________

If you were interacting *with VANA students* on your unit during the past year, please <u>only answer about those students for questions 13-16</u>. Otherwise, answer about [University] nursing students in general present on your unit during the past year.

13. **On average, how much of the time were nursing students present on your unit during your shift(s) over the past year?**
    (Choose one)
    - ☐ All of the time
    - ☐ Most of the time
    - ☐ About half of the time
    - ☐ A little of the time
    - ☐ None of the time

14. **Of the time nursing students were present during your shift(s) over the past year, how much of that time were you interacting with those students?** Interactions might include precepting, supervising patient care, and work-related discussions.
    (Choose one)
    - ☐ All of the time
    - ☐ Most of the time
    - ☐ About half of the time
    - ☐ A little of the time
    - ☐ None of the time

15. **Thinking about the nursing students you have interacted with over the past year, how would you rate those students overall in the following areas?** If you do not have enough information to evaluate students in a particular area (e.g., you did not observe it, students don't perform that activity on your unit), check "Don't know."

	Above Average	Average	Below Average	Don't Know
	Choose one on each line			
Learning and utilizing the VA electronic medical record system (i.e. CPRS)	☐	☐	☐	☐
Understanding and delivering veteran-specific care (e.g., treatment of combat-related health conditions such as PTSD)	☐	☐	☐	☐
Understanding and following unit operational procedures	☐	☐	☐	☐
Working and communicating with unit staff	☐	☐	☐	☐
Initiative	☐	☐	☐	☐
Dependability	☐	☐	☐	☐

16. **Again, thinking about your experience with nursing students over the past year, in general, how satisfied or dissatisfied were you with the following?** If you feel that you do not have enough information or experience to answer, mark 'Don't know'.

	Very satisfied	Somewhat satisfied	Neither satisfied nor dissatisfied	Somewhat dissatisfied	Very dissatisfied	Don't Know
	Choose one on each line					
Information provided to you about students' learning objectives	☐	☐	☐	☐	☐	☐
Amount of time available for you to work with students	☐	☐	☐	☐	☐	☐
Support you received from supervisors/ colleagues to work with students	☐	☐	☐	☐	☐	☐
Preceptor-to-student ratio	☐	☐	☐	☐	☐	☐
Instructor-to-student ratio	☐	☐	☐	☐	☐	☐
Instructor involvement in teaching students while on the unit	☐	☐	☐	☐	☐	☐
Aptitude of students	☐	☐	☐	☐	☐	☐
Professional recognition you received for working with students	☐	☐	☐	☐	☐	☐
Personal reward from working with students	☐	☐	☐	☐	☐	☐
Teaching ability of instructors	☐	☐	☐	☐	☐	☐
Clinical expertise of instructors	☐	☐	☐	☐	☐	☐

These next questions ask about nursing students in an accelerated BSN program vs. those in a traditional BSN program with whom you were interacting on your unit during the past year.

17. **During the past year, were you interacting with nursing students in an accelerated BSN program?** If you cannot distinguish accelerated BSN students from traditional BSN students check "not sure."
    (Choose one)
    - ☐ Yes ➨ **GO TO Question 19**
    - ☐ No 3/7/2012 11:50:00 AM
    - ☐ Not sure

18. **Thinking about all nursing students on your unit during the past year, which best describes how they impacted your work?**
    (Choose one)
    - ☐ Made your work more difficult ➨ **GO TO Question 20**
    - ☐ Did not impact the difficulty of your work ➨ **GO TO Question 20**
    - ☐ Made your work less difficult ➨ **GO TO Question 20**

19. **Which best describes the impact that the following types of nursing students had on your work over the past year?**

	Made your work more difficult	Did not impact the difficulty of your work	Made your work Less difficult	No such students on this unit
	Choose one on each line			
Nursing students in an accelerated BSN program	☐	☐	☐	
Nursing students in a traditional BSN program	☐	☐	☐	☐

We now have a VANA-specific question.

20. **To what extent do you feel informed about the VANA program?**
    (Choose one)
    - ☐ Well informed
    - ☐ Somewhat informed
    - ☐ Not very informed

Next, we have a few questions about your education and professional training.

21. Which of the following degrees do you have? (Choose all that apply)

22. Did you receive this degree in nursing, in another area, or both? (Choose one on each line)

Degree (21)	Nursing	Another area	Both
☐ Doctoral degree ➡	☐	☐	☐
☐ Master's degree ➡	☐	☐	☐
☐ Bachelor's degree ➡	☐	☐	☐
☐ Associate's degree ➡	☐	☐	☐

23. What nursing credentials do you currently hold?

(Choose all that apply)

- ☐ Registered nurse (RN)
- ☐ Advanced practice nurse (APRN) with or without certification
- ☐ Clinical Nurse Specialist (CNS)
- ☐ Clinical Nurse Leader (CNL)
- ☐ Licensed Practical/Vocational Nurse (LPN/LVN)
- ☐ Nursing Diploma
- ☐ Other (Specify): ____

24. Are you currently enrolled or are you considering enrolling within the next two years, in any of the following higher education programs?

(Choose all that apply)

- ☐ Doctoral degree program in nursing
- ☐ Master's degree program in nursing
- ☐ Neither enrolled nor considering enrolling in any higher education program at this time ➡ **GO TO Question 28**
- ☐ Other educational program (nursing or other) (Specify): _________________________

25. Is the educational program that you are enrolled in or considering enrolling in at [University]?

(Choose one)

- ☐ Yes
- ☐ No

26. Where are you now in this process?
(Choose all that apply)

- ☐ Currently enrolled in a higher education program
- ☐ Have applied or in the process of applying to a higher education program
- ☐ Considering enrolling in a higher education program
- ☐ Other circumstances (Specify): ____

27. Has your VA's participation in VANA had any influence on your decision to pursue this higher degree or enroll in this educational program?
(Choose one)

- ☐ Yes
- ☐ No

We are almost at the end of the survey!

This next question asks about your satisfaction with your current VA position.

28. Currently, how satisfied are you with the following aspects of your work at the VA?

	Very satisfied	Somewhat satisfied	Neither satisfied nor dissatisfied	Somewhat dissatisfied	Very dissatisfied
	Choose one on each line				
Relationships with co-workers	☐	☐	☐	☐	☐
Opportunities for promotion	☐	☐	☐	☐	☐
Workload	☐	☐	☐	☐	☐
Work schedule	☐	☐	☐	☐	☐
Supervisors	☐	☐	☐	☐	☐

Lastly, we would like to ask a few general questions about you.

29. What is your current age?

(Choose one)

- ☐ Under 25
- ☐ 26-35
- ☐ 36-45
- ☐ 46-55
- ☐ Over 55

30. What is your gender?

(Choose one)

- ☐ Male
- ☐ Female

You have reached the end of our survey--thank you very much for completing it! Please feel free to share any other thoughts or recommendations. If necessary, you may continue your comments on the back of this paper.

FINAL INSTRUCTIONS

Please place your completed survey in the UPS envelope or box provided.
Thank you again for your participation!

Tamar Wyte, DPT, MPH
Project Director for the National Evaluation of the VA Nursing Academy
(818) 891-7711 x7940
tamar.wyte@va.gov
